# Comparison of the effects of cold water immersion and percussive massage on the recovery after exhausting eccentric exercise: A three-armed randomized controlled trial

**DOI:** 10.3389/fphys.2024.1432009

**Published:** 2024-09-23

**Authors:** Lars Heinke, Sasha Javanmardi, Ludwig Rappelt, Andreas Konrad, Robert Schleip, Axel J. Knicker, Jürgen Freiwald, Christian Baumgart

**Affiliations:** ^1^ Department of Movement and Training Science, University of Wuppertal, Wuppertal, Germany; ^2^ Department of Intervention Research in Exercise Training, German Sport University Cologne, Cologne, Germany; ^3^ Institute of Human Movement Science, Sport and Health, University of Graz, Graz, Austria; ^4^ Department of Sport and Health Sciences, Technical University of Munich, Munich, Germany; ^5^ Department of Health & Psychology, Diploma University of Applied Sciences, Bad Sooden-Allendorf, Germany; ^6^ Institute of Movement and Neurosciences, German Sport University Cologne, Cologne, Germany; ^7^ Research Center for Elite Sport, momentum, German Sport University Cologne, Cologne, Germany

**Keywords:** performance recovery, cold water immersion, percussive massage, fatigue, eccentric exercise, counter movement jumps, regeneration

## Abstract

**Introduction:**

Athletic training requires both challenging stimuli for adaptation and sufficient recovery for improved performance. While cold water immersion (CWI) is already a popular recovery method, handheld percussive massage (PM) devices have also gained popularity in recent years. This study aims to assess the effects of CWI and PM on performance recovery after strenuous eccentric exercises compared to a passive rest (PR) control condition.

**Methods:**

Thirty-four healthy physically active participants (9 females, 25 males) were randomly divided into three groups: CWI (n = 11), PM (n = 11), and passive rest (PR) (n = 12). They underwent an exhausting eccentric exercise protocol and different measurements at six time points (baseline, POST1, POST2, POST24, POST48, and POST72) over the time course of 72 h. These included subjective assessments of muscle soreness and perceived stiffness as well as measures of skin temperature, leg volume, creatine kinase activity, and three different jump tests. The eccentric exercise protocol consisted of 15 min downhill running (slope: 12%, speed: 10 km/h) and 3 sets of successive depth jumps (dropping height: 0.5 m) until individual exhaustion. After POST1 measurements, participants received 12 min of either CWI (11 ± 0.5°C), PM (40 Hz) or PR (supine posture).

**Results:**

No significant group effects were found for the number of depth jumps performed during the exhaustion protocol. All jump tests displayed a significant group × time interaction effect. Post-hoc analysis indicated significant lower jump heights in ΔPOST2 between CWI and both PM and PR. No other significant group effects were observed at any time point. No significant group × time interaction effects were noted for CK, leg volume, and soreness. The perceived stiffness showed a significant group × time interaction effect. Post-hoc analysis revealed a significant decrease in stiffness for PM compared to PR at ΔPOST2.

**Conclusion:**

Neither CWI nor PM showed any significant improvement in performance recovery over the 72-h period following strenuous eccentric exercise compared to PR. CWI showed an immediate performance decline which may be attributed to a cold-related reduction in motor nerve conduction velocity.

## 1 Introduction

Athletic training typically involves an overloading stimulus to induce adequate adaptions of the organism, but also adequate recovery is needed to enhance athletic performance as a result of training (Soligard et al., 2016). Training-induced muscular overload is capable of eliciting exercise-induced muscle damage (EIMD). In this context, particularly eccentric exercise is known to evoke prolonged subcellular muscle damage, pain and muscle weakness (Hedayatpour and Falla, 2015). Hence, achieving a balanced equilibrium between training load and recovery is essential to prevent an undesired training response, which can lead to a state of “Overreaching” or “Overtraining” (Meeusen et al., 2013) as well as to mitigate the risk of injuries and illnesses (Jones et al., 2017).

Athletes, coaches, and healthcare professionals use various post-exercise recovery methods to restore athletic performance swiftly. Cold water immersion (CWI) is widely recognized in various sports for its effectiveness, cost-efficiency, and ease of use as a method for performance recovery (Pournot et al., 2011). Current scientific evidence indicates that, compared to passive rest and other traditional recovery strategies, CWI leads to modest improvements in muscle soreness and functional deficits associated with EIMD (Leeder et al., 2012; Moore et al., 2022b; Xiao et al., 2023). Findings provided by Machado et al. (2016) also suggest that the beneficial effects of CWI depend on the dosage, with optimal recovery outcomes achieved at water temperatures ranging from 11°C to 15°C and application durations of 11–15 min.

Besides the positive effects of CWI on post-exercise performance recovery, the use of vibration treatments to alleviate EMID is controversially discussed throughout the literature. The meta-analysis of Lu et al. (2019) highlighted that applying local mechanical vibration directly to the muscle or tendon and engaging in whole-body vibration through vibrating platforms could positively influence performance recovery. Conversely, Pournot et al. (2016) found that a vibration therapy was not more effective than passive recovery. Additionally, Fuller et al. (2015) failed to confirm any positive effects of vibration therapy on performance recovery compared to a placebo. Much like the limited evidence supporting the effectiveness of vibration therapy, there is also a dearth of clear evidence to substantiate the efficacy of manual massage for performance recovery (Poppendieck et al., 2016). Despite the uncertain evidence, handheld percussive massage devices, often referred to as massage guns, have been steadily gaining popularity among athletes, coaches, and healthcare professionals (Cheatham et al., 2021). These devices combine elements from both manual percussive massage and vibration therapy. Cheatham et al. (2021) showed in their cross-sectional survey study that these devices are widely used among healthcare professionals with the aim of enhancing post-exercise recovery and to modulate pain. To date, only a few studies have investigated the acute performance recovery effects of a handheld percussive massage device (García-Sillero et al., 2021; Leabeater et al., 2023).

To the best of our knowledge, as of the present, there has been no study that has comprehensively assessed the effectiveness of handheld percussive massage (PM) treatment in contrast to established recovery strategies concerning post-exercise performance recovery. This gap in research is particularly noteworthy given the widespread use of these devices by coaches, athletes, and healthcare professionals, who currently need more relevant information on their efficacy.

Therefore, this study aimed to investigate the effects of a 12-min PM treatment of the lower extremities on performance recovery after exhausting prolonged and intermittent eccentric exercise compared to a time-matched CWI and a passive control condition. According to the controversial evidence on the effects of vibration therapy and massage and the evident beneficial results of cold water immersion, we hypothesized that the PM treatment is less effective for post-exercise performance recovery than CWI.

## 2 Materials and methods

### 2.1 Participants and study design

Thirty-four healthy physically active subjects (9 females, 25 males) participated in the study. A Meta-analysis of Leeder et al. (2012) suggested medium effects of CWI on muscular power recovery over the course of 72 h, while no reference data is available for PM effects. An a-priori sample size calculation using G*Power software (latest ver. 3.1.9.7; University of Düsseldorf, Germany) for a repeated measures within-between interaction model revealed the need of at least 21 participants ((α = 0.05, study power (1-β-error) = 0.80, r = 0.6, effect size ηp2 = 0.06 (f = 0.25)). Participants were randomized into either a CWI group (CWI, n = 11; female = 3, male = 8; age = 25.4 ± 2.6 years, body height = 1.78 ± 0.08 m, body mass = 74.4 ± 11.8 kg) a PM group (PM, n = 11; female = 3, male = 8; age = 23.7 ± 3.4 years, body height = 1.75 ± 0.10 m, body mass = 76.4 ± 14.5 kg) or a passive rest group (PR, n = 12; female = 3, male = 9; age = 23.9 ± 4.3 years; body height = 1.80 ± 0.10, body mass = 76.1 ± 12.4 kg) using a spreadsheet for allocating each subject to a group for treatment as soon as the subject was recruited (Hopkins, 2010) (Table 1). Participants were instructed to not perform any strenuous activity 48 h before the first day of testing and throughout the study duration. Before measurement, all participants were fully informed of the experimental procedures and provided their written informed consent. The study’s accordance with the Declaration of Helsinki was confirmed by the University ethics committee (MS/AE 211222).

**TABLE 1:** Mean values ± standard deviations for anthropometric and physiological data, performance indices and items of perceived rating of pain and stiffness for the cold water immersion (CWI), Percussive Massage (PM) and Passive rest (PR) groups at BASELINE. *p*-values and partial eta squared of the individually conducted analysis of variance (ANOVA) are also reported.

Baseline participant characteristics	CWI (n = 11)	PM (n = 11)	PR (n = 12)	*p*-value	η_p_^2^
Age (yrs)	25.4 ± 2.6	23.7 ± 3.4	23.9 ± 4.3	*p* = 0.50	0.04
Mass (kg)	74.4 ± 11.8	76.4 ± 14.5	76.1 ± 12.4	*p* = 0.92	0.00
Height (m)	1.78 ± 0.08	1.75 ± 0.10	1.80 ± 0.10	*p* = 0.57	0.04
Jumps (n)	125 ± 113	103 ± 67	99 ± 46	*p* = 0.70	0.02
Stiffness (VAS, a.u.)	28.5 ± 23.0	25.1 ± 24.3	15.3 ± 9.6	*p = 0.26*	0.08
Soreness (a.u.)	0.5 ± 1.0	0.3 ± 0.6	1.1 ± 1.2	*p = 0.15*	0.12
CK (units/L)	185 ± 77	210 ± 129	222 ± 127	*p = 0.73*	0.02
DJ RSI (ms^-1^)	1.20 ± 0.26	1.32 ± 0.31	1.43 ± 0.38	*p = 0.25*	0.08
SJ (cm)	29.4 ± 4.6	29.1 ± 5.9	28.0 ± 6.6	*p = 0.82*	0.01
CMJ (cm)	31.4 ± 4.6	31.9 ± 6.2	31.8 ± 6.7	*p = 0.98*	0.00
Leg volume (mL)	7,393 ± 1,028	7,558 ± 1,112	7,590 ± 1,098	*p = 0.90*	0.01

CK: creatine kinase, CMJ: Counter-Movement-Jump, DJ RSI: reactive strength index during drop jump, SJ: squat jump, VAS: visual analog scale.

The study was designed as a randomized controlled trial. Participants visited the laboratory five times, the first being a familiarization session at least 1 week before the exhaustion protocol. Furthermore, the measurements were conducted at baseline (baseline), immediately after the exhaustive protocol (POST1), immediately after the recovery intervention (POST2), and at the three subsequent days (POST24, POST48, POST72). All participants completed the same semi-individualized exhaustion protocol, the only difference being the post-exercise recovery treatment. To investigate the effects of CWI and percussive massage on post-exercise performance recovery, participants performed maximal squat jumps (SJ), countermovement jumps (CMJ), and drop jumps (DJ) from a dropping height of 0.3 m. Furthermore, subjective values of muscle soreness were reported, perceived stiffness was specified, thigh skin temperature and volume of the lower extremities were measured, and capillary blood samples were taken to determine creatine kinase activity.

### 2.2 Measurements

At each time point (baseline, POST1, POST2, POST24, POST48, and POST72), the participants performed three maximal SJ, three CMJ, and three DJ from a dropping height of 0.3 m. During the jumps, the hands were placed at the hips to limit upper body involvement in the assessment of jumping performance. The vertical jump height was calculated using the impulse–momentum (SJ and CMJ) or flight time method (DJ) (Linthorne, 2001). Vertical ground reaction forces were recorded at a sampling rate of 1,000 Hz using two synchronized rigid force plates (400 × 600 mm; Type 9287BA, Kistler, Winterthur, Switzerland), one for each foot. The force plates were connected to a personal computer running a customized data acquisition software developed using LabView (version 9.0f2, National Instruments, Austin, United States). This software converted the raw electrical potential data (in V) from the force plates into force data (in N) along the x-, y-, and *z*-axes. The force data were then exported into a custom Excel (.xlsx) file for further analysis. During each test, participants were instructed to remain as still as possible on the force plates for a few seconds after each jump to ensure accurate data collection. Each series of three jumps was recorded in a single file. All subsequent data analysis was performed using R (version 4.0.5) in the integrated development environment RStudio (version 1.4.1106), utilizing customized algorithms to calculate the relevant performance metrics. The Reactive Strength Index (RSI) for the drop jumps (DJ) was computed by dividing the jump height by the ground contact time (Flanagan et al., 2008). At the same time points, subjective assessments of the muscle soreness and perceived stiffness were conducted. For evaluation of muscle soreness, a numeric rating scale ranging from 0 to 10, where 0 indicated no muscle soreness, and 10 indicated maximal soreness was used (Thompson et al., 1999). Participants were asked to compare their current level of soreness to their past sports experiences. “Soreness” was defined as their perceived muscle pain and any impairment in muscle function. Additionally, participants were instructed to assess their perceived muscle stiffness by evaluating feelings of muscle tightness or any perception of a reluctance to stretch the muscle group (Jones et al., 1987). Perceived stiffness of the lower extremities was measured using a Visual Analog Scale (VAS) ranging from 0 to 100 mm, with 0 indicating no stiffness and 100 indicating maximum perceived stiffness. Skin temperature was measured using an infrared camera (PI 640i, Optris GmbH, Berlin, Germany). The volume of the lower extremities was assessed using the Perometer 350 T (Pero-System GmbH, Wuppertal, Germany). This device measures the maximum horizontal and vertical diameters at 4.7 mm intervals along the limb’s longitudinal axis, and calculates segmented and total volumes based on these measurements. The Perometer’s reliability in assessing lower limb volume has been demonstrated by Man et al. (2004). At baseline, and at 24, 48, and 72 h post-exercise, 10 μL capillary blood samples were collected from the earlobe to measure creatine kinase (CK) activity using the SimplexTAS 101 Analyzer (Sysmex Europe SE, Norderstedt, Germany). CK activity was determined through a coupled reaction where creatine phosphate and ADP are converted to creatine and ATP. This reaction leads to the reduction of NADP + to NADPH, which is measured photometrically at 340 nm. The amount of NADPH produced is directly proportional to the CK activity in the blood. The measurements were consistently carried out in the following sequence: muscle soreness, CK levels, 5-min warm-up, skin temperature, leg volume, perceived stiffness, and jump tests (SJ, CMJ, DJ).

#### 2.2.1 Missing values report

For the POST72 measurements, it is essential to note that the dataset for one participant is incomplete due to technical reasons. The measurements for this particular participant were not recorded.

### 2.3 Exhaustion protocol

The exhaustion protocol was applied during the second visit with baseline measurements, POST1 and POST2. After a 5-min standardized cycle ergometer warm-up (80 W, 80 rpm), participants performed baseline measurements followed by a 15-min downhill run on a treadmill. The slope was set to −12% and the speed to 10 km/h, respectively. The prolonged exercise was immediately followed by 3 sets of successive depth jumps (Verkhoshansky, 1967) from a dropping height of 0.5 m. A resting period of 1 min was observed between sets. To maximize muscular fatigue and, consequently, increase the range of eccentric motion during the initial landing of each depth jump—an aspect that can precede muscle damage—participants were instructed to land as “smoothly” as possible and rebound as high as possible with each jump. Within each of the first two sets of depth jumps, each participant performed 20 depth jumps. In the final set, participants continued jumps until they could not maintain at least 90% of the mean jump height of the first sets in 3 consecutive jumps (Comyns et al., 2011).

### 2.4 Recovery treatment

After finishing POST1 measurements, participants received either CWI, PM, or PR recovery treatment. The CWI consisted of 12 min with the lower extremities immersed up to hip height in a bucket filled with cold water maintained at 11°C ± 0.5°C by the addition of crushed-ice as needed. During PM, participants were placed in a supine position. The same investigator consistently applied the percussive massage treatment using a Hypervolt 2 device (Hyperrice, California, United States). The massage was conducted with the hardball attachment head, provided by the manufacturer at a frequency of 40 Hz. The duration of PM was in terms of total treatment time matched to the CWI. More precisely, the PM was applied to the muscles of the left and right calf for 4 min and to the muscles of the left and right thigh for 8 min. The investigator started the PM at the medial side of the treated muscle and moved the device longitudinally in a straight line from distal to proximal and back to distal within 20 s. Back at the distal end of each muscle, the investigator moved the device laterally and repeated the described procedure (Konrad et al., 2020). The investigator always tried to apply the same pressure to the skin. To steer the applied pressure, participants were asked to give verbal feedback on the perceived pressure rated on a scale ranging from 0 to 10, where 0 represented no pressure and 10 unendurable pressure. The applied pressure should be experienced as “moderate” (4) to “medium” (6). During PR, participants were placed in a supine position for 12 min, while no further treatments were applied.

### 2.5 Statistics

All data are presented as mean ± standard deviation (sd). All outcome measures were initially checked for normal distribution (visual inspection of QQ-plots and Shapiro-Wilk-tests) and variance homogeneity (Levene-Test). To examine possible baseline differences in group characteristics and protocol (i.e., number of jumps performed during the fatigue protocol), analyses of variance (ANOVA; group: CWI vs. PR vs. PM) were performed separately. For all further analyses, the respective differences for all outcome measures between baseline and POST1, POST2, POST24, POST48, and POST72 were calculated (ΔPOST1, ΔPOST2, ΔPOST24, ΔPOST48, and ΔPOST72), with values > 0 indicating higher values compared to baseline. Thereon, individually conducted 3 (group: CWI vs. PR vs. PM) × 5 (time: ΔPOST1, ΔPOST1, ΔPOST24, ΔPOST48, ΔPOST72) repeated measures of variance (rANOVA) were performed. Mauchly’s sphericity test was performed, and Greenhouse-Geisser (GG) corrections were applied if necessary. Effect sizes for rANOVA are given as partial eta squared (ηp2) with ≥0.01, ≥0.06, ≥0.14 indicating small, moderate, and large effects, respectively (Cohen, 1988). Subsequently, in case of significant interaction effects, Bonferroni *post hoc* tests were computed. All statistical analyses were performed using R (version 4.0.5) in its integrated development environment, RStudio (version 1.4.1106). For all statistical analyses, a *p*-value below 0.05 was considered statistically significant.

## 3 Results

### 3.1 Fatigue protocol and baseline differences

**FIGURE 1:**
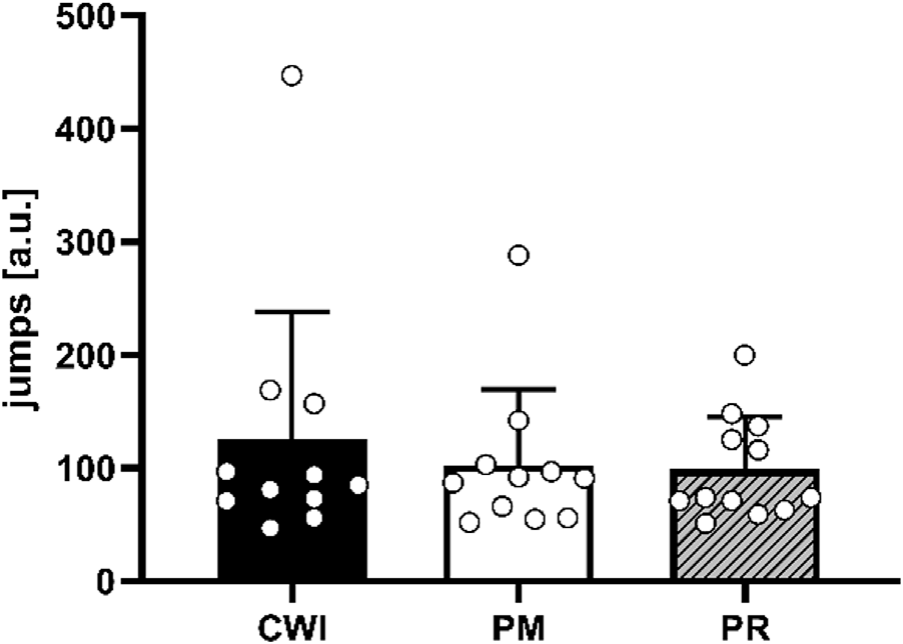
Mean values and standard deviations for the number of jumps performed by the CWI (black), the PM (white), and PR (lines) groups during the fatigue protocol. Individual values are indicated by white dots.

No baseline differences were found in anthropometric data, nor in any of the measured parameters (see Table 1). Moreover, no significant “group” effects were found for the number of jumps performed during the fatigue protocol (F (2, 31) = 0.36, p = 0.70, ηp2 = 0.02) (Figure 1).

### 3.2 Jumping performance differences

**FIGURE 2:**
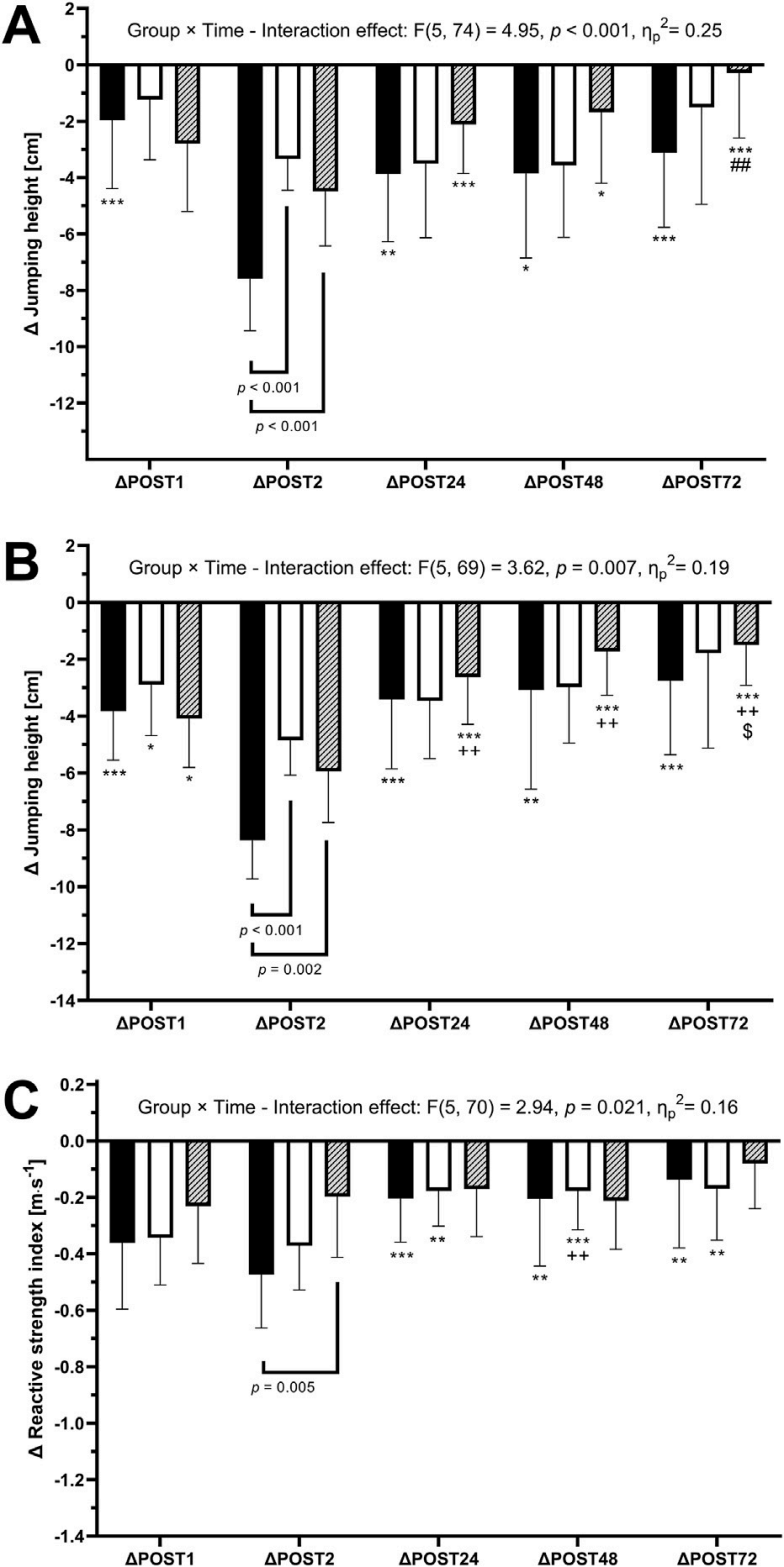
Differences to baseline values (mean value ±standard deviation)in jumping performance during Squat Jump **(A)**, Counter-Movement-Jump **(B)** and Drop-Jump (C; Drop-Jump Reactive Strength Index) for CWI (black), the PM (white), and PR (lines) groups for the timepoints immediately after the depth jump protocol (POST1), immediately after the acute recovery intervention (POST2) as well as 24h, 48h and 72 h after the depth jump protocol (POST24, POST48 and POST72, respectively). Between-group differences are indicated by brackets, within-group differences by signs. *: significantly larger than POST2 (*p* < 0.05), **: significantly larger than POST2 (*p* < 0.01), ***: significantly larger than POST2 (*p* < 0.001),. ##: significantly larger than POST48 (*p* < 0.01), ++: significantly larger than POST1 (*p* < 0.01), $: significantly larger than POST24 (*p* < 0.05).

For SJ, a statistically significant lower performance was found for ΔPOST2 of CWI compared to both PM and PR (p < 0.001). Furthermore, in CWI, a statistically significant lower performance was found for ΔPOST2 compared to all other time points (p ≤ 0.010). Similarly, in PR, significant differences were found for ΔPOST2 compared to ΔPOST24 – ΔPOST72 (p ≤ 0.018) and for ΔPOST48 compared to ΔPOST72 (p = 0.004) (Figure 2A).

For CMJ statistically significant lower performance was found for ΔPOST2 of CWI compared to both PM and PR (*p* ≤ 0.001). Furthermore, in CWI, a statistically significant lower performance was found for ΔPOST2 compared to all other time points (*p* ≤ 0.002). Similarly, in PR, significant differences were found between ΔPOST2 and all other time points (*p* ≤ 0.041), between ΔPOST1 and ΔPOST24 – ΔPOST72 (*p* ≤ 0.023) and for ΔPOST24 compared to ΔPOST72 (*p* = 0.003). Additionally, CMJ height showed significant differences for ΔPOST2 compared to ΔPOST1 in PM (*p* = 0.033) (Figure 2B).

For DJ RSI, a significant differences between CWI and PM for ΔPOST2 (*p* < 0.01) was found. Moreover, in CWI, significant differences were found between ΔPOST2 compared to ΔPOST24 – ΔPOST72 (*p* ≤ 0.005). Similarly, in PR, significant differences were found between ΔPOST2 and ΔPOST24 – ΔPOST72 (*p* ≤ 0.007). Also, in PR, ΔPOST1 showed significant differences compared to ΔPOST48 (*p* = 0.033) (Figure 2C).

### 3.3 Temperature and CK and leg volume

For both CK and leg volume, no significant group × time interaction effects, but large and significant effects for the main analysis of time were revealed (F (2, 48) = 9.68, *p* < 0.001, η_p_^2^ = 0.24 and F (3, 79) = 21.6, *p* < 0.001, η_p_^2^ = 0.43). For CK, both CWI and PM showed significantly lower differences to baseline at ΔPOST72 compared to ΔPOST24 (CWI: *p* < 0.001, PM: *p* = 0.009) and ΔPOST48 (CWI: *p* < 0.001, PM: *p* = 0.002) with also significant differences between ΔPOST24 and ΔPOST48 in CWI (*p* < 0.001). For leg volume, in CWI, pairwise comparison between time points showed a large effect, indicating a significantly lower leg volume at POST2 compared to all other time points for all groups (Table 2).

**TABLE 2:** Differences (mean value ± standard deviation) to baseline values of skin temperature, creatine kinase and leg volume immediately after the depth jump protocol (ΔPOST1), immediately after the acute recovery intervention (ΔPOST2) as well as 24h, 48h and 72 h after the depth jump protocol (ΔPOST24, ΔPOST48 and ΔPOST72, respectively). Furthermore, repeated measure of variance (rANOVA) *p*-values and partial eta squared (η_p_^2^) are reported.

Outcomes	Group	ΔPOST1	ΔPOST2	ΔPOST24	ΔPOST48	ΔPOST72	rANOVA *p*-value (η_p_^2^)		
							Time	Group	Time × group
Temperature (°C)	CWI	−0.4 ± 1.7^***^	−11.3 ± 2.1^#^	−0.6 ± 1.2^***^	−0.1 ± 1.2^***^	−0.4 ± 1.0^***^	<0.001 (0.22)	<0.001 (0.68)	<0.001 (0.82)
	PR	−0.2 ± 1.6^**^	2.3 ± 1.6	−0.6 ± 1.4^*^	−0.4 ± 0.9^*^	−0.2 ± 0.8^**^			
	PM	−0.7 ± 2.3^***^	2.3 ± 1.4	−0.3 ± 1.4^*^	−0.2 ± 2.0	−0.3 ± 1.4^*^			
Creatine Kinase (units/L)	CWI	-	-	479 ± 198	188 ± 115	93 ± 107	<0.001 (0.24)	0.40 (0.06)	0.16 (0.11)
	PR	-	-	484 ± 573	566 ± 852	270 ± 661			
	PM	-	-	781 ± 667	353 ± 272	162 ± 144			
Leg volume (mL)	CWI	−36 ± 79	−74 ± 77	33 ± 94	64 ± 190	50 ± 130	<0.001 (0.43)	0.66 (0.03)	0.51 (0.06)
	PR	−16 ± 110	−103 ± 99	49 ± 81	63 ± 89	80 ± 90			
	PM	−50 ± 96	−62 ± 91	80 ± 136	73 ± 120	71 ± 162			

*Significantly different from ΔPOST2 of the same group (*p* < 0.05), **significantly different from ΔPOST2 of the same group (*p* < 0.01), ***significantly different from ΔPOST2 of the same group (*p* < 0.001), #significantly different from ΔPOST2 of all other groups (*p* < 0.001).

### 3.4 Perceived soreness and stiffness

The rANOVA revealed a large and significant group × time interaction effect for VAS (F (5, 80) = 2.78, *p* = 0.02, η_p_^2^ = 0.16). Post-hoc testing revealed significant differences between PM and PR for ΔPOST2 (*p* = 0.048). Moreover, significant differences were found between ΔPOST72 and both ΔPOST24 and ΔPOST48 in all three groups (*p* ≤ 0.024) (Table 3).

**TABLE 3:** Differences to baseline values (mean value ±standard deviation) of the perceived stiffness (VAS) and the perceived soreness immediately after the depth jump protocol (ΔPOST1), immediately after the acute recovery intervention (ΔPOST2) as well as 24h, 48h and 72 h after the depth jump protocol (ΔPOST24, ΔPOST48 and ΔPOST72, respectively). Furthermore, repeated measure of variance (rANOVA) *p*-values and partial eta squared (η_p_^2^) are reported.

Outcomes	Group	ΔPOST1	ΔPOST2	ΔPOST24	ΔPOST48	ΔPOST72	rANOVA *p*-value (η_p_^2^)		
							Time	Group	Time × group
Stiffness (VAS, a.u.)	CWI	31.5 ± 21.4^#^	27.4 ± 25.2	37.1 ± 22.0^**, #^	30.1 ± 26.9^*, ##^	1.5 ± 20.8	<0.001 (0.34)	0.56 (0.04)	0.02 (0.16)
	PR	34.8 ± 26.5	33.0 ± 22.2	39.3 ± 23.2^*^	36.5 ± 26.6^*^	19.7 ± 25.6			
	PM	28.6 ± 26.2	9.4 ± 18.8	39.8 ± 27.8^*^	45.7 ± 25.9^**^	12.9 ± 21.7			
Soreness (a.u.)	CWI	-	-	6.6 ± 1.8	7.0 ± 1.1	3.6 ± 1.7	<0.001 (0.63)	0.08 (0.15)	0.71 (0.03)
	PR	-	-	5.6 ± 2.4	5.7 ± 2.8	3.3 ± 2.8			
	PM	-	-	7.3 ± 1.8	7.7 ± 1.3	5.0 ± 1.9			

*Significantly different from ΔPOST72 of the same group (*p* < 0.05), **significantly different from ΔPOST72 of the same group (*p* < 0.01), ^#^significantly different from ΔPOST2 of the same group (*p* < 0.05), ^##^significantly different from ΔPOST2 of the same group (*p* < 0.01).

For soreness, no significant group × time interaction effect was found (F (4, 60) = 0.53, *p* = 0.71, η_p_^2^ = 0.03). However, large and significant effects for the main analysis of time were revealed (F (2, 60) = 51.7, *p* < 0.001, η_p_^2^ = 0.63) with considerable difference in perceived soreness between ΔPOST72 and all other time points in all three groups (Table 3).

## 4 Discussion

This randomized-controlled study investigates the effects of a 12-min percussive massage treatment (PM) of the lower extremities on performance recovery after exhausting prolonged and intermittent eccentric exercise compared to a time-matched cold water immersion (CWI) and a passive control condition (PR). The primary findings of this study were as follows: (I) Neither CWI nor PM resulted in a significant improvement in performance recovery over a 72 h period following exhaustive eccentric exercise when compared to passive rest (PR). (II) PM resulted in an immediate adjustment, specifically an acute reduction, in the subjective perception of stiffness compared to PR. However, this effect was not associated with a simultaneous improvement in performance parameters during acute recovery. (III) CWI caused an immediate reduction in jumping performance.

Percussive massage as a combination of conventional massage and vibration therapy is thought to provide beneficial effects on performance recovery. It is considered to include the advantages of conventional massage, such as alleviating muscle tension, reducing muscle pain, swelling, and spasms, improving muscle blood flow, and facilitating the removal of substances like blood lactate or creatine kinase (Best et al., 2008). Moreover, it is regarded as incorporating the effects of vibration therapy, such as inhibiting pain sensory receptors (Kosar et al., 2012). We observed an immediate reduction (POST1: 28.6 ± 26.2 to POST2: 9.4 ± 18.8) in perceived stiffness after the PM treatment. This observation could possibly be attributed to decreased residual cross-bridges, as mechanical agitation of a muscle can potentially disrupt them (Hill, 1968). However, compared to PR, we did not detect any improvement in performance recovery parameters after the treatment. These findings align with the results of Leabeater et al. (2023), who similarly observed no impact on the immediate recovery of physical measures (ankle range of motion, calf circumference, isometric strength, calf endurance) following intense calf exercise. The authors employed a 5-min session of calf percussive massage using a hand-held device to address the restoration of these physical measures. In contrast to our findings and the results reported by Leabeater et al. (2023), García-Sillero et al. (2021) observed acute performance recovery effects when using PM as an inter-set performance recovery tool during a 3-min break between 4 sets of bench press exercises. Therefore, it seems plausible that differences in the nature of exercise (e.g., eccentric vs. concentric) may differently affect the acute effect of PM as a recovery tool. This is in line with the findings of Jönhagen et al. (2004) regarding the effects of massage on performance recovery. In their study, sixteen subjects underwent 300 maximal eccentric quadriceps contractions on both legs. One leg received daily massage while the other served as a control over 3 days. Despite using massage as a recovery tool, no significant impact was observed on the level or duration of pain, as well as on the loss of strength and muscle function during the 3 days following intense eccentric exercise. Contrary, Lau and Nosaka (2011) employed five sessions of 30-min of hand-held vibration massage over the course of four consecutive days to assess strength performance recovery in the arms, resulting in minor positive effects. Nevertheless, while the treatment effectively reduced delayed-onset muscle soreness and enhanced range of motion recovery, no significant effects were observed on objective performance recovery markers such as blood lactate, creatine kinase, or C-reactive protein levels. However, it is crucial to consider that Lau and Nosaka (2011) utilized untrained subjects in their study. Recovery treatments might be more effective in this population due to potential psychological influences, such as a positive effect on the perception of recovery and mood enhancement, as indicated in the Weerapong et al. (2005) review. In light of previous findings and our results concerning recovery within 24–72 h following exhaustive exercise, we suggest that a single 12-min percussive massage treatment may be insufficient to improve performance recovery significantly.

Our study results showing no improvement in performance recovery after CWI compared to PM or PR. Therefore, it can be suggested that the one-time CWI application might not be sufficient to induce adequate performance recovery in our participants. Numerous meta-analytical studies, including Leeder et al. (2012), Choo et al. (2022), and Moore et al. (2022a), have supported the potential of cold water immersion (CWI) in mitigating muscle damage effects, evidenced by reduced creatine kinase levels, alleviation of delayed onset muscle soreness, and improved perceived recovery. However, studies measuring dynamic muscle power are limited. Within these, the most favorable results were observed in those employing the most extended total duration of CWI application over a 72 h period. Vaile et al. (2008) demonstrated a noteworthy enhancement in squat jump peak power compared to passive recovery through 14 min of CWI at 15°C, administered every 24 h across 72 h following seven sets of 10 repetitions of eccentric bilateral leg press contractions.

Furthermore, we observed a notable decline in jumping performance immediately after the CWI application, aligning with findings previously demonstrated by Wiewelhove et al. (2018). This phenomenon can be attributed to the impact of cold application on nerve conduction parameters, as demonstrated by Herrera et al. (2010). According to their findings, CWI has a notable effect on latency and motor nerve conduction velocity, leading to increased latency and decreased nerve conduction velocity. We hypothesized that such modulation of nerve conduction parameters contributed to our study’s observed decline in jumping performance. Furthermore, lowering tissue temperature not only substantially diminishes the contractile force of muscle fibers under a consistent neural drive (Pedersen et al., 2003) but also, separately, increases the stiffness of associated collagenous tissues (Hasberry and Pearcy, 1986) impairing the elongation capacities of the tissue. These dual effects will likely contribute to a decline in jumping performance, even when nerve conduction velocity remains unaltered. As per our findings, we do not recommend CWI as a short-term recovery method, specifically for immediate effects on performance parameters lasting up to a few minutes.

Moreover, several factors may contribute to the absence of the expected effects of CWI over the 72-h measurement period. The ability of CWI to reduce post-exercise muscle soreness and inflammation is often attributed to the constriction of blood vessels (Moore et al., 2022a). However, it is important to critically assess the role of inflammation in muscle repair during recovery. As highlighted by Peake et al. (2017), CWI did not significantly affect the exercise-induced infiltration of inflammatory cells. Furthermore, the study reported an increase in mRNA expression of pro-inflammatory cytokines and neurotrophins, as well as the subcellular translocation of heat shock proteins in muscle tissue following CWI application. While a certain level of inflammation is necessary for muscle regeneration and adaptation, as it prompts the secretion of anti-inflammatory molecules such as IL-10, IL-12, and IL-1ra (Fatouros and Jamurtas, 2016), our findings seems to align with those of Peake et al. (2017). This study suggests that CWI may not effectively minimize the inflammatory and stress response in muscle after resistance exercise. Although we did not measure inflammatory markers other than CK activity, we speculate that CWI did not significantly modulate the inflammatory response in our study, which could have interfered with the body’s natural healing processes. Consequently, CWI showed no improvement in performance recovery compared to PR. Furthermore, despite our adherence to the CWI treatment parameters observed in the review on the dose-response relationship by Machado et al. (2016), we have to consider that not everyone responds to CWI in the same way. Indeed, as demonstrated by Paulsen et al. (2012), there is a generalizable high inter-subject variability in the response to eccentric exercise. This variability can result in a wide range of outcomes, including mild to severe performance declines, varying levels of creatine kinase activity, and differences in inflammation levels. Therefore, it is crucial to consider that individuals may respond differently to CWI when interpreting our results.

While this study offers valuable insights for practical application and suggests potential directions for future research, it is essential to acknowledge some limitations in the study design that need to be addressed. The study’s findings may have limited generalizability due to the specific nature of the exercise-induced exhaustion used in the research. The study utilized exhaustive eccentric exercise, and it is suggested that different types of exercises and exercise-induced fatigue may yield different results (Poppendieck et al., 2016). This specificity in the exercise protocol could limit the applicability of the findings to a broader range of athletic activities and conditions. Therefore, the study’s findings may only partially represent the effectiveness of PM and CWI in various exercise contexts. Furthermore, we must highlight the high inter-subject variability in response to performance recovery treatments. It acknowledges that different individuals may respond differently to various recovery treatments, especially if considering other training states (Weerapong et al., 2005), making it challenging to generalize its effectiveness across all populations.

In conclusion, our study found that neither CWI nor PM significantly improved performance recovery compared to PR over the 72-h period following strenuous eccentric exercise. CWI led to an immediate performance decline, likely due to a cold-induced reduction in motor nerve conduction velocity. This observation raises questions about the usefulness of CWI as a short-term recovery method, particularly for its immediate effects on performance. Although PM significantly reduced perceived stiffness at POST2 compared to PR, this did not translate into improvements in other performance parameters. Our findings suggest that PM may not deliver the desired outcomes for which it is commonly used by healthcare professionals and athletes, as highlighted by Cheatham et al. (2021). The lack of significant overall improvements with both CWI and PM in our study highlights the variability in individual responses to recovery methods, as shown in previous research. Given the subjective nature of perceived recovery, the absence of consistent positive effects in our data suggests that a one-size-fits-all approach to using CWI or PM may not be suitable. Athletes should consider their personal responses and preferences when selecting performance recovery strategies. Future research should prioritize individualized recovery approaches to optimize performance and reduce the risk of overuse injuries.

## Data Availability

The raw data supporting the conclusions of this article will be made available by the authors, without undue reservation.
